# Methyl 4-(trimethyl­silylethyn­yl)benzoate

**DOI:** 10.1107/S1600536808008192

**Published:** 2008-05-03

**Authors:** Storm Potts, Dinabandhu Das, Martin W. Bredenkamp

**Affiliations:** a Department of Chemistry and Polymer Science, University of Stellenbosch, Private Bag X1, Matieland, 7602, South Africa

## Abstract

The title compound, C_13_H_16_O_2_Si, was synthesized as a precursor for ethynylarene derivatives and crystallized from hexane. In the crystal structure, mol­ecules are linked by weak C—H⋯O hydrogen bonds to form chains that pack in layers in a herringbone fashion.

## Related literature

For related literature, see: Eddaoudi *et al.* (2001 ▶); Dybtsev *et al.* (2004 ▶); Kesanli *et al.* (2005 ▶); Zhao *et al.* (2004 ▶); Allen *et al.* (1987 ▶); Fasina *et al.* (2005 ▶).
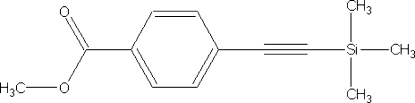


## Experimental

### 

#### Crystal data

C_13_H_16_O_2_Si
*M* *_r_* = 232.35
Orthorhombic, 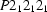

*a* = 6.1983 (11) Å
*b* = 7.1194 (12) Å
*c* = 29.530 (5) Å
*V* = 1303.1 (4) Å^3^
*Z* = 4
Mo *K*α radiation
μ = 0.16 mm^−1^
*T* = 100 (2) K
0.25 × 0.24 × 0.08 mm

#### Data collection

Bruker APEX CCD area-detector diffractometer
Absorption correction: multi-scan (*SADABS*; Bruker, 2002 ▶) *T*_min_ = 0.960, *T*_max_ = 0.987
8160 measured reflections
3050 independent reflections
2643 reflections with *I* > 2σ(*I*)
*R*_int_ = 0.054

#### Refinement

*R*[*F* ^2^ > 2σ(*F* ^2^)] = 0.058
*wR*(*F* ^2^) = 0.114
*S* = 1.10
3050 reflections
149 parameters
H-atom parameters constrained
Δρ_max_ = 0.37 e Å^−3^
Δρ_min_ = −0.30 e Å^−3^
Absolute structure: Flack (1983 ▶), 1136 Friedel pairs
Flack parameter: −0.01 (19)

### 

Data collection: *SMART* (Bruker, 2002 ▶); cell refinement: *SAINT* (Bruker, 2003 ▶); data reduction: *SAINT*; program(s) used to solve structure: *SHELXS97* (Sheldrick, 2008 ▶); program(s) used to refine structure: *SHELXL97* (Sheldrick, 2008 ▶); molecular graphics: *X-SEED* (Barbour, 2001 ▶; Atwood & Barbour, 2003 ▶); software used to prepare material for publication: *publCIF* (Westrip, 2008 ▶).

## Supplementary Material

Crystal structure: contains datablocks I, New_Global_Publ_Block. DOI: 10.1107/S1600536808008192/ez2120sup1.cif

Structure factors: contains datablocks I. DOI: 10.1107/S1600536808008192/ez2120Isup2.hkl

Additional supplementary materials:  crystallographic information; 3D view; checkCIF report

## Figures and Tables

**Table 1:** Hydrogen-bond geometry (Å, °)

*D*—H⋯*A*	*D*—H	H⋯*A*	*D*⋯*A*	*D*—H⋯*A*
C11—H11*A*⋯O1^i^	0.98	2.58	3.470 (4)	151
C12—H12*A*⋯O1^ii^	0.98	2.57	3.527 (3)	167

Symmetry codes: (i) 
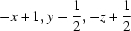
; (ii) 
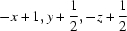
.

**Fig. 1.**
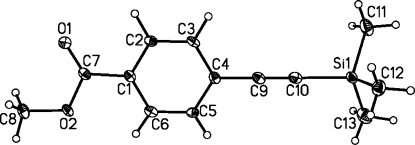
The molecular structure of the title compound showing the atom-labelling scheme. Displacement ellipsoids are shown at the 50% probability level.

**Fig. 2.**
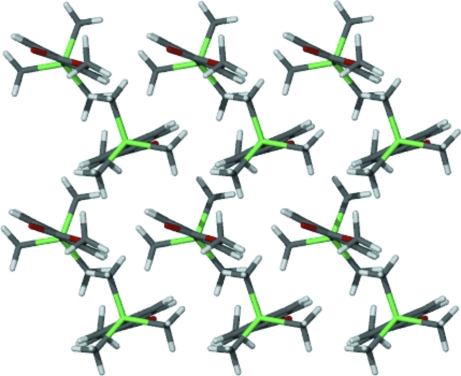
Herring-bone arrangement of the molecules, viewed down the c axis.

**Fig. 3.**
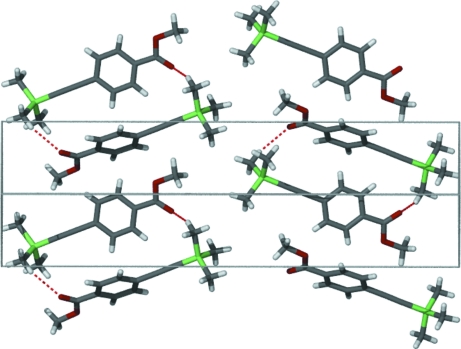
C—H···O hydrogen bonds formed between a methyl hydrogen of the trimethylsilyl group and a neighbouring carbonyl oxygen atom. Hydrogen bonds are shown as dashed lines.

## supplementary crystallographic information

### Comment

The title compound was isolated as a precursor in the synthesis of a series of ethynylarene-based ligands with terminal carboxylate groups. Interest in these kinds of ligands can be attributed to their ability to incorporate metal ions into M—O—C clusters, leading to novel metal-organic frameworks (MOFs), a category of compounds gaining increasing interest due to their potential applications for gas storage and separation and catalysis (Eddaoudi *et al.*, 2001; Dybtsev *et al.*, 2004; Kesanli *et al.*, 2005; Zhao *et al.*, 2004). The structure of the title compound (I) is shown in Fig. 1. Molecules of (I) pack in layers parallel to the (010) plane forming herring-bone motifs (Fig. 2). Analysis of the crystal packing shows that the molecules are arranged in alternating directions in the layer, due to the bulky trimethylsilyl groups facilitating the close packing of the molecules with the adjacent layer along the *c* axis. The methyl hydrogen atoms of the trimethysilyl group form C—H···O hydrogen bonds with the carbonyl oxygen atom on the adjacent molecule (Fig. 3).

The acetylenic bond distance [C9—C10 1.200 (3) Å] corresponds with the average value detailed in Allen *et al.* (1987) for C*_sp_*≡ C*_sp_*–C_sp2_ (Ar).

### Experimental

The title compound (I), was synthesized from trimethylsilylacetylene and 4-iodo(methylbenzoate) using a Sonogashira cross-coupling-type reaction as detailed in (Fasina *et al.*, 2005). Recrystallization from hexane afforded crystals of the title compound.

^1^H and ^13^C NMR spectra were recorded as an additional method of characterization, ^1^H NMR (CDCl_3_, 400 *MHz*): *δ* = 0.22 (9*H*, s, SiCH_3_), 3.89 (3*H*, s, CO_2_CH_3_), 7.49–7.53 (2*H*, m, ArH), 7.95–7.99 (2*H*, m, ArH); ^13^C-NMR (CDCl_3_, 75.5 MHz): *δ* = -0.44 (SiCH_3_), 52.063 (OCH_3_), 97.738 (CC), 104.16 (CC), 127.952 (ArH), 129.53 (ArH), 129.896 (ArH), 132.029 (ArH), 166.761 (CO)

### Refinement

Hydrogen atoms were refined in calculated positions, using a riding model (C–H = 0.98–0.99 Å, *U*_iso_(H) = 1.5*U*_eq_(C) for methyl C or 1.2*U*_eq_(C) or the remaining C atoms).

### Crystal data

**Table:**

C_13_H_16_O_2_Si	*F*_000_ = 496
*M**_r_* = 232.35	*D*_x_ = 1.184 Mg m^−^^3^
Orthorhombic, *P*2_1_2_1_2_1_	Mo *K*α radiation λ = 0.71073 Å
Hall symbol: P2ac2ab	Cell parameters from 1456 reflections
*a* = 6.1983 (11) Å	θ = 2.8–23.3º
*b* = 7.1194 (12) Å	µ = 0.16 mm^−^^1^
*c* = 29.530 (5) Å	*T* = 100 (2) K
*V* = 1303.1 (4) Å^3^	Plate, colourless
*Z* = 4	0.25 × 0.24 × 0.08 mm

### Data collection

**Table:**

Bruker APEX CCD area-detector diffractometer	3050 independent reflections
Radiation source: fine-focus sealed tube	2643 reflections with *I* > 2σ(*I*)
Monochromator: graphite	*R*_int_ = 0.054
*T* = 100(2) K	θ_max_ = 28.3º
/w scans	θ_min_ = 2.8º
Absorption correction: multi-scan(SADABS; Bruker, 2002)	*h* = −7→8
*T*_min_ = 0.960, *T*_max_ = 0.987	*k* = −8→9
8160 measured reflections	*l* = −37→32

### Refinement

**Table:**

Refinement on *F*^2^	Hydrogen site location: inferred from neighbouring sites
Least-squares matrix: full	H-atom parameters constrained
*R*[*F*^2^ > 2σ(*F*^2^)] = 0.058	*w* = 1/[σ^2^(*F*_o_^2^) + (0.0431*P*)^2^ + 0.2577*P*] where *P* = (*F*_o_^2^ + 2*F*_c_^2^)/3
*wR*(*F*^2^) = 0.114	(Δ/σ)_max_ < 0.001
*S* = 1.10	Δρ_max_ = 0.37 e Å^−^^3^
3050 reflections	Δρ_min_ = −0.30 e Å^−^^3^
149 parameters	Extinction correction: none
Primary atom site location: structure-invariant direct methods	Absolute structure: Flack (1983), 1136 Friedel pairs
Secondary atom site location: difference Fourier map	Flack parameter: −0.01 (19)

### Special details

**Table:**

Geometry. All e.s.d.'s (except the e.s.d. in the dihedral angle between two l.s. planes) are estimated using the full covariance matrix. The cell e.s.d.'s are taken into account individually in the estimation of e.s.d.'s in distances, angles and torsion angles; correlations between e.s.d.'s in cell parameters are only used when they are defined by crystal symmetry. An approximate (isotropic) treatment of cell e.s.d.'s is used for estimating e.s.d.'s involving l.s. planes.
Refinement. Refinement of *F*^2^ against ALL reflections. The weighted *R*-factor *wR* and goodness of fit *S* are based on *F*^2^, conventional *R*-factors *R* are based on *F*, with *F* set to zero for negative *F*^2^. The threshold expression of *F*^2^ > σ(*F*^2^) is used only for calculating *R*-factors(gt) *etc*. and is not relevant to the choice of reflections for refinement. *R*-factors based on *F*^2^ are statistically about twice as large as those based on *F*, and *R*- factors based on ALL data will be even larger.

### Fractional atomic coordinates and isotropic or equivalent isotropic displacement parameters (Å^2^)

**Table:**

	*x*	*y*	*z*	*U*_iso_*/*U*_eq_	
Si1	0.68188 (11)	0.55394 (11)	0.42983 (2)	0.01481 (17)	
O1	0.1541 (3)	0.5852 (3)	0.12946 (6)	0.0236 (5)	
O2	−0.1514 (3)	0.4974 (3)	0.16417 (6)	0.0182 (4)	
C1	0.1563 (4)	0.5421 (4)	0.20957 (8)	0.0138 (5)	
C2	0.3623 (4)	0.6175 (4)	0.21449 (9)	0.0138 (5)	
H2	0.4368	0.6646	0.1888	0.017*	
C3	0.4582 (4)	0.6241 (4)	0.25659 (9)	0.0146 (5)	
H3	0.5981	0.6770	0.2598	0.017*	
C4	0.3507 (4)	0.5533 (4)	0.29466 (8)	0.0133 (5)	
C5	0.1428 (4)	0.4787 (4)	0.28942 (8)	0.0137 (5)	
H5	0.0673	0.4322	0.3151	0.016*	
C6	0.0467 (4)	0.4724 (3)	0.24702 (8)	0.0138 (5)	
H6	−0.0936	0.4206	0.2436	0.017*	
C7	0.0588 (4)	0.5439 (4)	0.16347 (8)	0.0145 (5)	
C8	−0.2620 (4)	0.5084 (4)	0.12112 (9)	0.0220 (7)	
H8A	−0.2696	0.6397	0.1113	0.033*	
H8B	−0.4084	0.4581	0.1244	0.033*	
H8C	−0.1831	0.4347	0.0985	0.033*	
C9	0.4551 (4)	0.5535 (4)	0.33826 (8)	0.0149 (5)	
C10	0.5448 (4)	0.5509 (4)	0.37428 (8)	0.0169 (5)	
C11	0.9744 (4)	0.5113 (5)	0.42078 (10)	0.0299 (8)	
H11A	0.9953	0.3856	0.4080	0.045*	
H11B	1.0505	0.5202	0.4498	0.045*	
H11C	1.0318	0.6055	0.3998	0.045*	
C12	0.6371 (5)	0.7888 (4)	0.45560 (9)	0.0219 (6)	
H12A	0.6989	0.8858	0.4360	0.033*	
H12B	0.7068	0.7937	0.4854	0.033*	
H12C	0.4819	0.8108	0.4591	0.033*	
C13	0.5661 (5)	0.3652 (4)	0.46578 (10)	0.0272 (7)	
H13A	0.4092	0.3806	0.4676	0.041*	
H13B	0.6281	0.3728	0.4962	0.041*	
H13C	0.5999	0.2426	0.4525	0.041*	

### Atomic displacement parameters (Å^2^)

**Table:**

	*U*^11^	*U*^22^	*U*^33^	*U*^12^	*U*^13^	*U*^23^
Si1	0.0106 (3)	0.0178 (4)	0.0160 (3)	0.0025 (3)	−0.0022 (3)	−0.0019 (3)
O1	0.0155 (9)	0.0366 (12)	0.0188 (10)	−0.0016 (10)	0.0001 (8)	0.0049 (9)
O2	0.0108 (9)	0.0249 (11)	0.0189 (9)	−0.0047 (8)	−0.0051 (7)	0.0009 (7)
C1	0.0119 (11)	0.0130 (13)	0.0166 (12)	0.0010 (12)	−0.0024 (9)	−0.0018 (11)
C2	0.0115 (12)	0.0129 (13)	0.0171 (14)	0.0011 (10)	0.0019 (10)	0.0008 (10)
C3	0.0085 (12)	0.0145 (13)	0.0208 (14)	−0.0016 (10)	−0.0010 (10)	−0.0032 (11)
C4	0.0119 (11)	0.0115 (12)	0.0164 (12)	0.0039 (12)	−0.0024 (9)	−0.0016 (11)
C5	0.0127 (12)	0.0123 (13)	0.0162 (12)	−0.0025 (11)	0.0024 (9)	0.0015 (10)
C6	0.0115 (12)	0.0086 (13)	0.0213 (13)	0.0009 (10)	0.0001 (10)	−0.0019 (11)
C7	0.0124 (11)	0.0110 (12)	0.0199 (13)	0.0007 (11)	−0.0024 (10)	0.0001 (12)
C8	0.0181 (14)	0.0266 (17)	0.0212 (14)	−0.0029 (11)	−0.0079 (11)	−0.0028 (12)
C9	0.0134 (11)	0.0112 (12)	0.0202 (13)	−0.0005 (12)	−0.0010 (10)	−0.0013 (12)
C10	0.0122 (12)	0.0167 (14)	0.0218 (14)	−0.0009 (12)	0.0016 (10)	−0.0021 (12)
C11	0.0186 (14)	0.0392 (19)	0.0320 (18)	0.0086 (13)	−0.0050 (12)	−0.0188 (14)
C12	0.0231 (16)	0.0255 (16)	0.0171 (15)	0.0053 (12)	−0.0037 (12)	−0.0011 (12)
C13	0.0223 (16)	0.0280 (17)	0.0313 (18)	0.0039 (13)	−0.0063 (13)	0.0061 (14)

### Geometric parameters (Å, °)

**Table:**

Si1—C10	1.848 (3)	C5—H5	0.9500
Si1—C13	1.857 (3)	C6—H6	0.9500
Si1—C11	1.858 (3)	C8—H8A	0.9800
Si1—C12	1.858 (3)	C8—H8B	0.9800
O1—C7	1.202 (3)	C8—H8C	0.9800
O2—C7	1.344 (3)	C9—C10	1.200 (3)
O2—C8	1.447 (3)	C9—C4	1.441 (3)
C1—C6	1.390 (3)	C11—H11A	0.9800
C1—C2	1.392 (3)	C11—H11B	0.9800
C1—C7	1.490 (3)	C11—H11C	0.9800
C2—H2	0.9500	C12—H12A	0.9800
C3—C2	1.379 (4)	C12—H12B	0.9800
C3—H3	0.9500	C12—H12C	0.9800
C4—C3	1.401 (3)	C13—H13A	0.9800
C4—C5	1.403 (3)	C13—H13B	0.9800
C5—C6	1.387 (3)	C13—H13C	0.9800
			
C10—Si1—C13	108.75 (14)	O1—C7—O2	123.3 (2)
C10—Si1—C11	108.62 (12)	O1—C7—C1	124.5 (2)
C13—Si1—C11	109.95 (15)	O2—C7—C1	112.2 (2)
C10—Si1—C12	107.78 (13)	Si1—C11—H11A	109.5
C13—Si1—C12	111.06 (14)	Si1—C11—H11B	109.5
C11—Si1—C12	110.61 (14)	H11A—C11—H11B	109.5
C7—O2—C8	115.63 (19)	Si1—C11—H11C	109.5
C10—C9—C4	178.7 (3)	H11A—C11—H11C	109.5
C3—C4—C5	119.0 (2)	H11B—C11—H11C	109.5
C3—C4—C9	120.2 (2)	O2—C8—H8A	109.5
C5—C4—C9	120.8 (2)	O2—C8—H8B	109.5
C6—C1—C2	120.2 (2)	H8A—C8—H8B	109.5
C6—C1—C7	122.1 (2)	O2—C8—H8C	109.5
C2—C1—C7	117.7 (2)	H8A—C8—H8C	109.5
C2—C3—C4	120.4 (2)	H8B—C8—H8C	109.5
C2—C3—H3	119.8	Si1—C12—H12A	109.5
C4—C3—H3	119.8	Si1—C12—H12B	109.5
C3—C2—C1	120.2 (2)	H12A—C12—H12B	109.5
C3—C2—H2	119.9	Si1—C12—H12C	109.5
C1—C2—H2	119.9	H12A—C12—H12C	109.5
C9—C10—Si1	178.4 (3)	H12B—C12—H12C	109.5
C6—C5—C4	120.4 (2)	Si1—C13—H13A	109.5
C6—C5—H5	119.8	Si1—C13—H13B	109.5
C4—C5—H5	119.8	H13A—C13—H13B	109.5
C5—C6—C1	119.8 (2)	Si1—C13—H13C	109.5
C5—C6—H6	120.1	H13A—C13—H13C	109.5
C1—C6—H6	120.1	H13B—C13—H13C	109.5
			
C5—C4—C3—C2	−1.1 (4)	C2—C1—C6—C5	0.2 (4)
C9—C4—C3—C2	177.5 (2)	C7—C1—C6—C5	−178.3 (2)
C4—C3—C2—C1	0.7 (4)	C8—O2—C7—O1	−2.8 (4)
C6—C1—C2—C3	−0.2 (4)	C8—O2—C7—C1	175.9 (2)
C7—C1—C2—C3	178.3 (2)	C6—C1—C7—O1	−172.3 (3)
C3—C4—C5—C6	1.1 (4)	C2—C1—C7—O1	9.2 (4)
C9—C4—C5—C6	−177.5 (2)	C6—C1—C7—O2	9.0 (4)
C4—C5—C6—C1	−0.6 (4)	C2—C1—C7—O2	−169.5 (2)

### Hydrogen-bond geometry (Å, °)

**Table:**

*D*—H···*A*	*D*—H	H···*A*	*D*···*A*	*D*—H···*A*
C11—H11A···O1^i^	0.98	2.58	3.470 (4)	151
C12—H12A···O1^ii^	0.98	2.57	3.527 (3)	167

Symmetry codes: (i) −*x*+1, *y*−1/2, −*z*+1/2; (ii) −*x*+1, *y*+1/2, −*z*+1/2.
